# Chinese medical students reflections on medical professionalism: A qualitative thematic analysis

**DOI:** 10.1097/MD.0000000000034640

**Published:** 2023-08-11

**Authors:** You-Yang Wang, Chuheng Chang, Wen Shi, Xiao-Ming Huang, Yang Jiao

**Affiliations:** a Department of General Practice (General Internal Medicine), Peking Union Medical College Hospital, Chinese Academy of Medical Sciences and Peking Union Medical College, Beijing, China; b Department of Gastroenterology, Peking Union Medical College Hospital, Chinese Academy of Medical Sciences and Peking Union Medical College, Beijing, China.

**Keywords:** 4 + 4 medical education, China, medical students, professionalism

## Abstract

There is no common medical professionalism framework in China, mandating work to conceptualize professionalism from various perspectives. Studies on students viewpoints about medical professionalism are limited. Therefore, this study aimed to investigate how Chinese medical students perceive professionalism to provide a reference for future medical education reform and policy development. Fifty-four written reflections on medical professionalism were collected from first-year students of China 4 + 4 medical education program enrolled in 2020 to 2021 academic years. Essays were subjected to thematic analysis using NVivo 12. Three main themes emerged: inter-personal, intra-personal, and public professionalism. Students emphasized the importance of physician-patient relationships, proficiency of medical knowledge, and enthusiasm for promoting health-related issues. By contrast, teamwork and confidentiality were not considered essential aspects of professionalism. The medical professionalism framework articulated by students in China was roughly the same as in other countries. Where there were differences, these may have been due to the unique sociocultural environment. Future medical professionalism education should be adjusted according to students understanding of professionalism.

## 1. Introduction

Medical professionalism describes a set of characteristics that guide physicians to serve patients, and it forms the cornerstone of high-quality medical education and clinical practice.^[1]^ Professionalism evolves in the context of geography and culture.^[2,3]^ Although medical professionalism is well discussed in the context of many non-western cultures and countries,^[4]^ there is no common consensus on the concept in mainland China. “*Medical Professionalism in the New Millennium: A Physician Charter”* (the “*Physician Charter*”) was introduced in China in 2005, but even ten years later students found it difficult to fulfill in practice.^[5]^ Even though the “*Chinese Medical Doctor Declaration”* was published in 2011, which integrated the *Physician Charter* with evolving Chinese societal changes,^[6]^ its acceptance is currently unknown. The current official declaration has not been widely recognized nor adopted, so it is important to investigate various perspectives on professionalism across the profession so that a consensus on this important concept can be reached.

Empirical studies on professionalism in China are also comparatively limited, especially with respect to students perspectives. The attributes defining medical professionalism are mainly theoretical and subjective, based on expert opinion.^[7]^ Pan et al^[8]^ used the nominal group technique with 6 Chinese medical students and found that clinical competence, humanism, and communication were the top 3 most important categories of professionalism, together with traditional cultural values, morality, teamwork, and self-management. Jiang et al^[9]^ explored medical students perceptions on the important attributes of good doctors, and found an overwhelming emphasis on ethics; however, “good doctors” are not necessarily equivalent to “professional doctors.”^[10]^

Therefore, the purpose of this study was to explore medical students perspectives on professionalism in mainland China to address knowledge gaps in the literature. Given that increasing patient-provider conflicts in China are partly attributable to some doctors exhibiting low levels of professionalism,^[11]^ this study aimed to improve our knowledge about professionalism in medical education to build better patient-provider relationships that will ultimately help to promote effective health system reform.

## 2. Methods

### 2.1. Setting

In China, medical education programs are 8 years long to train future physicians and medical scientists. There are 2 types of program: the 8-year consistent model and the 4 + 4 model.^[12]^ The former, offered by 14 medical schools, is for high school graduates,^[13]^ while the latter, a graduate-entry medical program offered by 3 medical schools, is for students with non-medical baccalaureate degrees. This study was conducted at a 4 + 4 medical program at Peking Union Medical College, a leading medical school in China. Peking Union Medical College was the first Chinese school to offer the 8-year consistent medical education program after its foundation in 1917, and it started the 4 + 4 program in 2018. The 4 + 4 program mainly recruits students from high ranked domestic and overseas universities and has a capacity of 30 to 40 students per year.^[14]^

### 2.2. Participants and data collection

All 30 first-year medical students enrolled in the 4 + 4 medical education program 2020 class and 32 in the 2021 class were invited to participate in this study at the beginning of their first semesters. These students had no prior professional experience, medical training, nor had they participated in formal courses in medical professionalism or medical ethics. Since individual written reflection is considered a useful source of data in qualitative research,^[15]^ students were asked to complete the following assignment: “Describe what medical professionalism means to you.”

### 2.3. Data analysis

Most students (n = 54, 36 females and 18 males, aged between 19 and 28, mean age 22) returned their reflection notes, an 87.1% response rate. Data were analyzed using thematic analysis, which generally followed the 6-step process identified by Braun and Clarke.^[16]^ Under the supervision of the lead researchers (XH, YJ), 2 researchers (YW, CC) read the reflection notes, independently generated initial codes, and wrote the first draft of the themes and sub-themes. During the process, computer-assisted qualitative data analysis software (NVivo 12, released 2018)^[17]^ was used to manage the data. After several discussions among research team members, the final themes and sub-themes were developed by consensus, and appropriate quotes were selected to illustrate themes or sub-themes. The analysis was a primarily inductive and data-driven process but also referenced existing research^[18,19]^ to define and name themes.

WS, XH, and YJ are all involved in medical student education and have long-term experience in teaching medical professionalism. YW and CC are students of the 4 + 4 medical education program and were trained in qualitative studies.

### 2.4. Ethical considerations

Written informed consent was obtained from all study participants. After participants consented to their essays being used, all identifiable information was removed to secure confidentiality. The Ethics Committee of Peking Union Medical College Hospital approved the study (S-K2075).

## 3. Results

Three main themes emerged from students perspectives of professionalism, namely: inter-personal professionalism; intra-personal professionalism; and public professionalism. The sub-themes represented by these 3 themes are shown in Table 1.

**Table 1:** Themes and subthemes.

Themes	Sub-themes
Interpersonal professionalism	Primacy of patient welfare
	Compassion and empathy
	Communication skills
Intrapersonal professionalism	Proficiency
	Lifelong learning
Public professionalism	Social justice
	Advocacy
	Professional reputation

### 3.1. Interpersonal professionalism

At the heart of professionalism, students tended to mention the doctor-patient relationship first. Interpersonal professionalism indeed reflects the care provided by doctors to patients.

#### 1.3.1. Primacy of patient welfare.

In students reflections, patient welfare was considered the essence of professionalism. Although the meaning of medical professionalism may sometimes differ slightly and vary between cultures, the primacy of patient welfare was believed to be universally accepted.

“As long as the career goal of doctors is to help people, primacy of patient welfare is always a guideline for medical professionalism… Doctors deal with precious and sacred life, and thus altruism casts the other pursuits into the shade.”

“When personal interests conflict with patient welfare, a doctor with good professional manners gives priority to the needs of patients. At the outbreak of COVID-19, Chinese doctors went to the frontline to support and treat critically ill patients without hesitation. They are really respectable and what they did gives a good interpretation of professionalism.”

Although students were willing to devote themselves to altruism, they were confused about how to balance pursuing individual interests and putting the best interests of patients first. One student wrote, “*There is a big gap between being completely altruistic and reality. I really hope that doctors can help patients with the assurance of their individual interests and welfare.”*

Another student wrote, “*Here is the scenario: a patient comes to ask for help when a doctor is off duty. Should the doctor treat the patient following the spirit of ‘patient first’ and sacrifice his own rest time? In this situation, doctors should also be guaranteed reasonable leisure time rather than be forced to work all day long.”*

#### 2.3.1. Compassion and empathy.

A tenet of “*to cure sometimes, to relieve often, to comfort always”* was frequently quoted to illustrate the desired qualities of compassion and empathy, within the context that some diseases are untreatable and medicine is limited.

“Doctors should also have a deep understanding of compassion and empathy, and put it into action when communicating with patients. If a doctor does not show loving care for patients, he is not qualified as a professional doctor, even if he is knowledgeable. Patients come to see a DOCTOR, not an emotionless MACHINE, so it is far from enough to provide a correct but cold diagnosis and treatment for patients.”

“In many situations, doctors cannot completely eliminate pain, cure the disease, or restore patients to health; they can only try to provide humanistic care. Doctors should put themselves in the patient’s shoes and recognize patients needs.”

#### 3.3.1. Communication skills.

Together with the attributes of compassion and empathy, communication skills are crucial to building trust in the patient-physician relationship. Communication is the vital medium for delivering compassion and empathy.

“Excellent communication skills are a necessary component of professionalism. Doctors should encourage patients to express themselves as well as keep the conversation focused. The minimum requirement for communication is to obtain information for diagnosis, but professional doctors can obtain more, such as trust, mutual understanding, and collaboration with patients.”

“Dealing with patients and their families requires an abundance of communication skills. Doctors should be able to explain diagnosis and treatment options in an easy-to-understand way to patients. Based on the premise of full communication, a patient’s right to shared decision-making can be fully realized. Treatment is not selling goods condescendingly, but rather to work, communicate, and cooperate with patients towards the common goal.”

### 3.2. Intrapersonal professionalism

Based on the conviction of “patient first,” students then emphasized the importance of intra-personal professionalism, because “*doctors mainly rely on clinical knowledge to meet the demands of helping patients*.” Students complained that “*The available documents about professionalism gave less attention to the firm grasp needed of medical knowledge or skills*.”

#### 1.3.2. Proficiency.

Almost all students described the need for a doctor to be proficient. Being skillful and excellent in clinical knowledge and skills were perceived as the foundation of professionalism.

“The primary duty of doctors is to treat diseases and relieve pain, for which proficiency is a significant guarantee. In most patients opinion, doctors are qualified and trustworthy if they can save lives, but unqualified if the opposite happens. Therefore, the first requirement of professionalism is to master sufficient medical knowledge and technique.”

“Proficiency should not be acknowledged as a low barrier to entry for professionalism; on the contrary, it is a major indictor for doctors to distinguish excellence from mediocrity. Hence, I think that doctors need to make improvement of medical knowledge and skills a priority.”

#### 2.3.2. Lifelong learning.

As medical sciences have advanced at a such a rapid pace, lifelong learning was also mentioned by students. Commitment to lifelong learning was consistent with maintaining proficiency.

“With advances in knowledge and technology in medicine, only by adhering to the attitude ‘keep on learning as long as you live’ can doctors avoid being outdated. Doctors really need to improve themselves over time so that they can apply the most advanced medical knowledge to treatment.”

“To ensure efficient treatment, doctors should not only master current knowledge and skills, but also have the desire for new knowledge and exploration, and keep in touch with the latest research.”

### 3.3. Public professionalism

Public professionalism was regarded as an advanced requirement, compared with interpersonal or intrapersonal professionalism. Students highlighted social justice, professional reputation, and advocacy in health-related issues outside direct clinical practice.

#### 1.3.3. Social justice.

Students perceived a lack of high-quality medical resources and their uneven distribution. To change this situation, distributing social and individual medical resources properly requires the effort of every doctor.

“Medical resources are relatively scarce, and how to distribute the limited resources is a huge problem for society. As direct allocators of medical resources, doctors should distribute healthcare resources fairly, regardless of patient race, religion, politics, gender, or income.”

“Medical resources in China are strained. For patients, making an appointment with a doctor in Peking Union Medical College Hospital is extremely difficult. For doctors, when he chooses to spend time on treating a patient, he actually gives up the chance to help other patients. The tension among patients is becoming fiercer than ever… It is a social problem that all doctors, or even the national medical system, should work on.”

#### 2.3.3. Advocacy.

There was widespread enthusiasm about advocating for public health individually or collectively. Public health issues, including providing health-related expertise to local populations, as well as political involvement, were frequently mentioned.

“In addition to completing daily work, doctors should also pay attention to the development of the entire medical service and public concerns. For some social issues, such as ‘too difficult to see a doctor and too expensive to seek healthcare’, doctors can solve them as frontline healthcare workers or policymakers after entering politics. Besides, there are many misunderstandings about health in our society. Doctors need to speak out for what is right in the spotlight, regardless of detractors.”

“Doctors can propose feasible suggestions about national or local medical policies and help patients address their problems to policymakers. In the case of public medical emergencies, doctors should more actively engage in the decision-making of the government. COVID-19 is a good example.”

#### 3.3.3. Professional reputation.

The relationship between individual doctors and medical groups was likened to a “*mapping relationship*.” For patients, the image of the entire medical group is represented by both the professional and unprofessional doctors they have met. Students thus reinforced the sense of collective honor and placed a high value on professional reputation.

“The personal behaviors of every doctor will affect patients and public judgment of the entire medical profession. Although the patient sees one doctor at a time, they use the experience to form a general image of physicians and fill in the blanks. One doctor’s unfriendly attitude is devastating enough to ruin the reputation of the hospital and even all doctors.”

“In China, most patients do not know their doctors before seeking medical advice. The previous stereotypes of doctors influence how they get on with other doctors in the future. If patients always contact doctors who are competent, considerable, and have excellent professionalism, it is easier for them to trust other doctors.”

## 4. Discussion

This study offers valuable insights into medical students perceptions of professionalism in mainland China. Moreover, to our knowledge, this is the first study on the Chinese 4 + 4 medical education program. We dissected students reflections and identified 3 main themes: inter-personal, intra-personal, and public professionalism. Students believed that the goal of professionalism was to help patients. To achieve this goal, doctors should be full of compassion and empathy, be good at communication and medicine, and actively participate in public-facing social work.

In terms of the reasons underpinning students perceptions of medical professionalism and the similarities and differences with Western students, our results indicated that medical students in mainland China and in other countries share many views on professionalism. Students enter medical school with spontaneous notions of professionalism,^[20]^ emphasizing the importance of doctor-patient relationships, which form the core of the social contract between doctors and society.^[21]^ Other prominent attributes identified in this study, such as communication skills, proficiency, and lifelong learning, were also extensively discussed by students in other countries.^[20,22–25]^ Meanwhile, medical students all expressed concerns about whether they should completely devote themselves to patients and worried about disruption to their private lives.^[26]^ Notably, Chinese students in this study also placed a high value on social responsibility, which can be regarded as progress, since Asian medical students used to be less enthusiastic about social justice and public service than Western students.^[9,27,28]^ There is also a persistent pessimistic view that young Asians are exposed to and overly driven by materialism and have become unconcerned with public issues.^[3]^

Notwithstanding these similarities, there were also some differences. “Teamwork” (with other medical professions) has less prominence in mainland China.^[22]^ Students may perceive this component as unimportant and unfamiliar because doctor-nurse relationships are still highly unbalanced^[29]^ and interprofessional education is still in its infancy in China.^[30]^ “Confidentiality” was also often ignored,^[23–25]^ possibly due to established stereotypes that Chinese society values privacy. However, patients expectations of privacy have become more prominent in China.^[31]^ This finding suggests that medical professionalism education should increase its focus on interprofessional cooperation and protection of patient privacy before these students confront real professionalism dilemmas in clinical settings. Although medical students in both Western countries and mainland China valued professional reputation,^[20,22]^ this aspect was more dominant in Chinese students reflections. This might be due to the sociocultural environment: there is a strong sense of collective honor in China,^[32]^ and therefore group members consciously protect the group reputation. In this case, Chinese students may have felt driven to protect the reputation and dignity of doctors, who are currently suffering from damage to their professional reputation.^[11]^ This emphasis on professional reputation may explain why medical students are more likely to drop out once they believe that the medical profession actually has a low social status and reputation.^[33]^

This study has several limitations. The study only involved first-year students from a single medical college. Given the importance of context in medical professionalism and that the focus on professionalism changes throughout training,^[34]^ the results may not be generalizable. This cohort of students studied in the 4 + 4 medical education program, but whether students in the 8-year consistent model and the 4 + 4 model have different views on professionalism remains uncertain. Further research in other contexts is needed to examine the universality of these findings. Qualitative methods also have inherent limitations. For example, the coding process in qualitative studies is inherently subjective, although the coders reached agreement in the process.

## 5. Conclusions

Analyzing written reflections from students provided an approach to understanding students expectations for medical professionalism. The perspectives of medical students in mainland China were broadly similar to other countries. However, these students placed more emphasis on the protection of professional reputation and less emphasis on teamwork and confidentiality. Medical educators should now tailor curricula and prioritize educational goals to focus on these characteristics of professionalism in the future.

## Author contributions

**Conceptualization:** Xiao-Ming Huang, Yang Jiao.

**Data curation:** You-Yang Wang, Chuheng Chang.

**Formal analysis:** You-Yang Wang, Chuheng Chang, Wen Shi, Xiao-Ming Huang, Yang Jiao.

**Investigation:** Wen Shi.

**Methodology:** You-Yang Wang, Chuheng Chang.

**Project administration:** You-Yang Wang, Chuheng Chang, Wen Shi, Yang Jiao.

**Resources:** Yang Jiao.

**Supervision:** Xiao-Ming Huang, Yang Jiao.

**Writing – original draft:** You-Yang Wang.

**Writing – review & editing:** Wen Shi, Xiao-Ming Huang, Yang Jiao.
